# Numerical simulation of high-temperature thermal contact resistance and its reduction mechanism

**DOI:** 10.1371/journal.pone.0194483

**Published:** 2018-03-16

**Authors:** Donghuan Liu, Jing Zhang

**Affiliations:** 1 Beijing Key Laboratory for Magneto-Photoelectrical Composite and Interface Science, School of Mathematics and Physics, University of Science and Technology Beijing, Beijing, P.R. China; 2 Basic Experimental Center for Natural Science, University of Science and Technology Beijing, Beijing, P.R. China; North China Electric Power University, CHINA

## Abstract

High-temperature thermal contact resistance (TCR) plays an important role in heat-pipe-cooled thermal protection structures due to the existence of contact interface between the embedded heat pipe and the heat resistive structure, and the reduction mechanism of thermal contact resistance is of special interests in the design of such structures. The present paper proposed a finite element model of the high-temperature thermal contact resistance based on the multi-point contact model with the consideration of temperature-dependent material properties, heat radiation through the cavities at the interface and the effect of thermal interface material (TIM), and the geometry parameters of the finite element model are determined by simple surface roughness test and experimental data fitting. The experimental results of high-temperature thermal contact resistance between superalloy GH600 and C/C composite material are employed to validate the present finite element model. The effect of the crucial parameters on the thermal contact resistance with and without TIM are also investigated with the proposed finite element model.

## Introduction

The heat-pipe-cooled leading edge concept is a potential option for reusable supersonic aircraft, and researchers had made many efforts on the theoretical, computational and experimental studies on this subject. Glass et al. proposed series of closed-form equations to evaluate heat-pipe-cooled leading edge design feasibility [1], and fabricated and tested Mo-Re heat pipes embedded in C/C structure with a D-shaped cross section, and found that the heat pipe did operate isothermally over a significant portion of its length [2–4]. Cao and Faghri proposed a complete mathematical model for transient two-dimensional high temperature heat pipes and investigated the transient responses of heat pipes to a pulsed heat input [5]. Wojicik and Clark investigated the performance of lithium filled heat pipe, they designed, fabricated and tested several refractory metal and niobium alloy heat pipes [6]. It is noted that all the previous studies had ignored the thermal contact resistance (TCR) between the inner heat pipe and outer heat resistive C/C composite material, which implies perfect thermal contact at the interface. The thermal protection mechanism of the-pipe-cooled leading edge requires that the aerodynamic heat must redistributed quickly from the stagnation area of the outer structure to the embedded heat pipes, while ignoring the thermal contact resistance between outer structure and inner heat pipe could possibly overestimate the heat transfer capability across the interface, which will cause potential safety problem to the whole structure.

**Fig 1:**
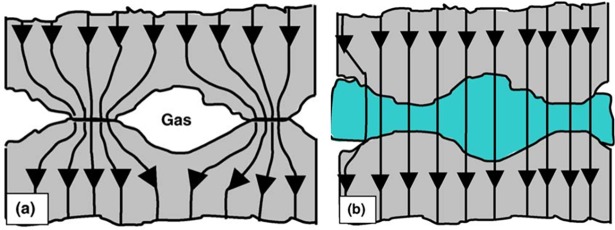
Effect of thermal interface material on the heat transfer at the interface [24]: (a) without interface material; (b) with interface material. The arrows denote the heat flow.

Thermal contact resistance exists in all the contact interface of various thermal structures due to the imperfect contact at the interface, since microscopic and macroscopic irregularities are present in all practical surfaces, and the actual area of contact for most metallic surfaces is only a small portion (about 1–2%) of the nominal contact area[7]. Thermal contact resistance has been widely studied in terms of theory, computation and experiment recently[8–16], while almost all the previous work mainly focus on low temperature or medium temperature (interface temperature less than 300°C), and interface temperature of the thermal protection structures of heat-pipe-cooled leading edges could reach as high as 500°C. At the same time, it is desirable for heat-pipe-cooled leading edges to reduce the interface thermal contact resistance, so that the stagnation heat can pass the structure interface easily to reach to the surface of the embedded heat pipe. One can reduce the thermal contact resistance with high contact pressures or smoother surfaces at the contact interface, while in many engineering applications, these options are limited, and using thermal interface material (TIM) could be the best choice[17]. In low and medium temperature environment, thermal grease[18], thermal pad[19], metal foil[20], low melting temperature alloys[21–23] etc. are always used as thermal interface materials, the mechanism of thermal interface material on reducing thermal contact resistance is illustrated[24] (Fig 1). In high temperature environment, soft carbon fiber and graphite sheet will be the ideal option for interface material.

It is not easy for the modeling of thermal contact resistance through both theoretical and experimental approaches, especially in high temperature environments, as material properties might be temperature-dependent, and radiative heat transfer through interface cavities is non-ignorable. Then finite element simulation of thermal contact resistance will be a useful supplement. A lot of methods for creating rough surfaces in the finite element model have been investigated. Prasanta and Niloy used 3D rough surfaces with a modified two-variable Weierstrass–Mandelbrot function with given fractal parameters in modeling real surfaces[25]. Hyun et al. created 3D surfaces by moving the coordinates of the finite element nodes by a fraction of the local height[26]. Jackson et al. presented a multi-scale model of thermal contact resistance between real rough surfaces that builds on Archard’s multi-scale description of surface roughness[27]. M.K. Thompson and J.M. Thompson discussed some of the benefits, techniques, challenges, and considerations associated with the incorporation of measured surfaces in finite element models[28]. Wilson et al. considered the multi-scale nature of surface roughness in a new model that predicts the real area of contact and surface separation as functions of load[29]. Sadowski and Stupkiewicz used the real roughness topographies obtained from 3D stylus profilometry measurements to model thermal contact resistance at high real contact area fractions[30]. It should be pointed out that these finite element modeling approaches are tedious and time-consuming, the important aspects such as temperature-dependent material properties and interface radiative heat transfer are always ignored which could cause significant error under high temperature environment. It is also noted that, another type of thermal resistance, Kapitza resistance, may exists at two perfect surfaces as well as carbon materials like graphene [31, 32]. Since the magnitude of the Kapitza resistance is round 10^−11^ m^2^K/W, compared with 10^−4^ m^2^K/W of the thermal contact resistance considered here, it is ignored in the present research.

The purpose of the present paper is to develop a compact finite element modeling approach based on the multi-point contact theory with experimental validation, and investigate the effect of the crucial parameters on the thermal contact resistance. Section 2 gives the details of the present numerical simulation approach, and Section 3 shows the whole process of the present approach through an example and discusses the effect of some crucial parameters on the thermal contact resistance. Conclusions are given in Section 4.

## Numerical simulation approach

### Multi-point contact model

From the microscopic point of view, real contact at the interface is primarily caused by the microscopic asperities of the real surface, and most of the heat through the interface flows through the actual contact spot. It is very important to modeling this actual contact as accurate as possible, which seems mission impossible in real circumstances. So many assumptions are introduced to make the theoretical analysis possible, such as single point contact model, also known as Holm tube model[33, 34], multi-point contact model[35, 36], and fractal model[37–39]. Numerical simulation related models can use surface profiles obtained from direct measurement of three dimensional surface geometry, although the geometry model is accurate, the requirement of large numbers of mesh elements and nodes make the numerical simulation nearly unfeasible, as the dimension discrepancy of the micro asperity and the macro structure is very huge, maybe five orders of magnitude. The present paper adopted the multi-point contact model in the numerical simulations for three reasons. Firstly, the geometry is simple and easy to achieve structured mesh which can greatly reduce the total finite element nodes of the model. Secondly, direct 3D surface scanning and reconstruction can be ignored which is time consuming. Thirdly, the geometry parameter of the micro-contact point can be determined by traditional measurement of surface roughness and a small quantity of thermal contact resistance experiments which can greatly improve the accuracy of the numerical model.

**Fig 2:**
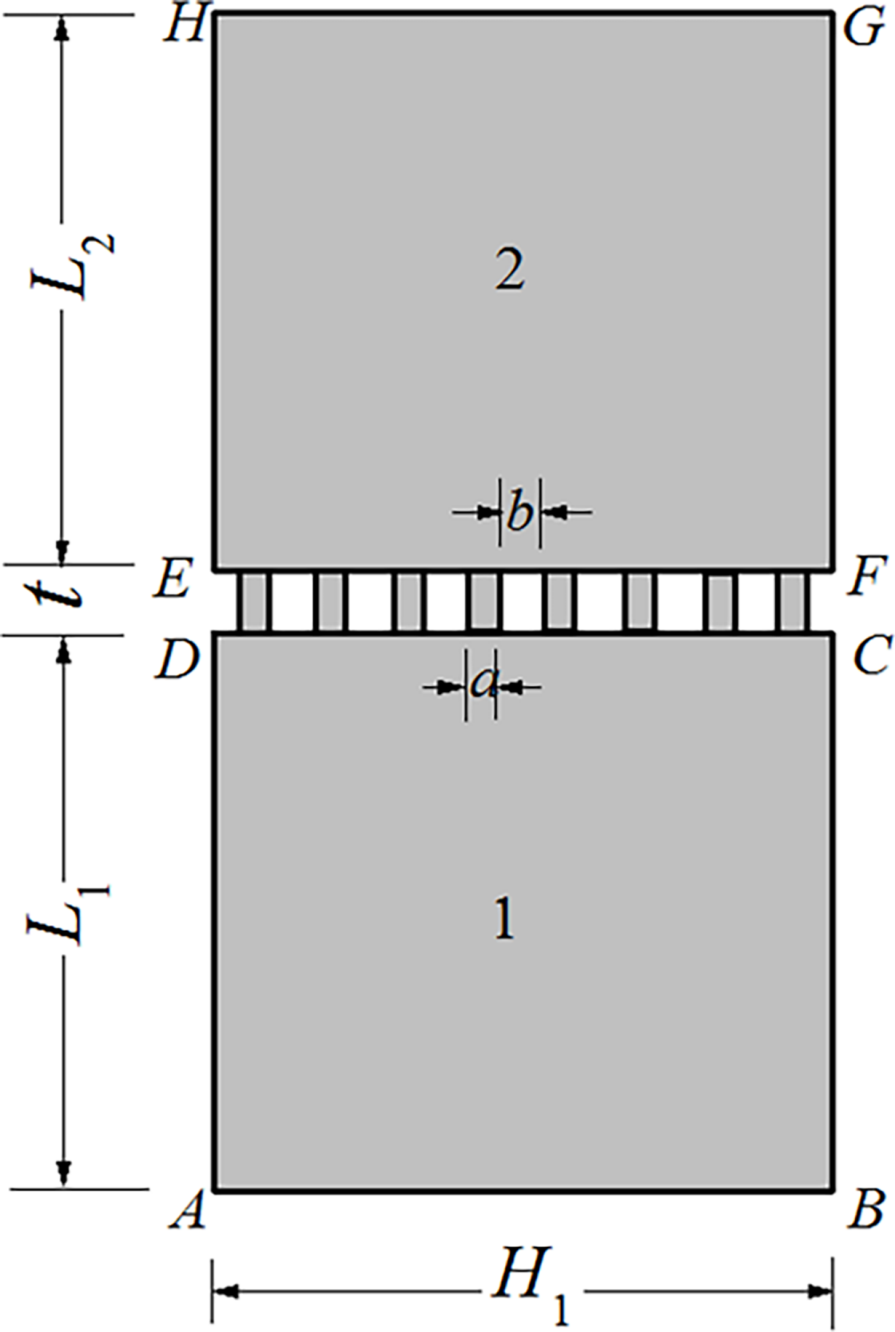
Computational model of the thermal contact resistance based on multi-point contact model.

For simplicity, due to the axial symmetry property of the experimental specimens, two-dimensional plane model is adopted here to model the real rough contact surface (Fig 2). The contact between two real surfaces is modeled by the contact between two perfect smooth surfaces with many micro-contact rectangles. The rectangular peaks are characterized by height *t* and width *a*, and the space between two neighbored peaks is *b*. *L*_1_ and *L*_2_ are the typical dimensions of the two contact specimens along the length direction, always several orders of magnitude larger than the dimension of the peak, and the width of the two specimens *H*_1_ are always identical. The determination of these parameters are given in the following subsection.

Compared with the single point contact model, the present multi-point contact model is more similar with the actual contact surface, at the same time, radiation and convective heat transfer through gaps between the micro-contact peaks can be taken into consideration. Through the adjustment of the spacing ratio of the micro-contact peaks, the effect of interface pressure can be considered, and the effect of interface material on reducing thermal contact resistance can also been take into account by modifying the equivalent thermal conductivity of the cavities between micro-contact peaks.

### Determination of model parameters

**Fig 3:**
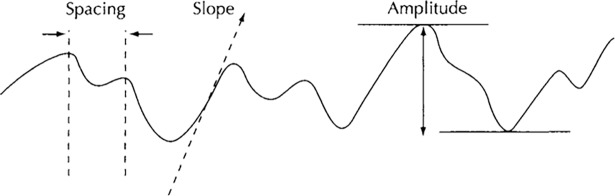
Surface profile characterization parameters [40].

It can be seen from Fig 2 that, the main geometric parameters to be determined are micro parameters *a*, *b* and *t*, and macro parameters *L*_1_, *L*_2_ and *H*_1_, and all these parameters are related with surface profile measurement and characterization. Three surface profile characterization parameters are always adopted in engineering practices[40] (Fig 3), and these are amplitude parameters, spacing parameters and hybrid parameters (slope).

For amplitude parameters, the *R*_a_ value is the universally recognized and widely used parameter of surface roughness, and it is the arithmetic mean of the magnitude of the deviation of the profile from the mean line. It is noted that *R*_a_ alone is not sufficient by itself to describe the roughness on a wide range of surfaces, since the same *R*_a_ value may corresponding to different periodic waveforms with very different peak-valley values[40], then the root-mean-squared roughness *R*_q_ could be a better alternative. In the present paper, the *R*_q_ value is adopted rather than *R*_a_ to characterize the height of the micro-contact rectangle, and the height of the micro-contact rectangle *t* is assumed the same as the equivalent roughness of the two contact surfaces given by:

$$t=\sqrt{R_{q1}^{2}+R_{q2}^{2}}$$ (1)

where *R*_q1_ and *R*_q2_ denote the root-mean-squared roughness of contact surface 1 and 2 respectively.

*S*_m_ is the most widely used spacing parameter, and it is the mean spacing between profile peaks at the mean line, measured within the sampling length, where a profile peak is the highest point of the profile between an upwards and downwards crossing of the mean line. Spacing parameters had been attracted less attention rather than the amplitude parameters in general engineering in the past. In contact circumstances, the amplitude parameters were considered to be most relevant since contact and wear always occurs at the peak of the surface, and spacing parameters had been concerned mainly in metallurgy and material science because of the need to examine the grain boundaries. In the numerical simulation of thermal contact resistance, spacing parameter of the microcontact is the key variable, as it decides the real contact area at the interface combined with the width of the micro-contact rectangle. Similar with the definition of the equivalent roughness, in the present paper, the spacing parameter *b* is given by:

$$b=\sqrt{S_{m1}^{2}+S_{m2}^{2}}$$ (2)

where *S*_m1_ and *S*_m2_ denote the mean spacing between profile peaks at the mean line of contact surface 1 and 2 respectively. It should be pointed out that, spacing *b* defined in Eq (2) corresponding to contact status with no interface pressure, while interface pressure increases, the spacing ratio becomes smaller, as the real contact area at the interface increases. As it is very difficult to give the analytical relationship between the interface pressure and spacing ratio *b*/*a*, the present paper determines this relationship through experimental data fitting, and this approach will be shown in Section 3. For simplicity, the width of the micro-contact rectangle *a* is set to be a constant with 10 *μ*m, as it always takes effect together with the spacing ratio *b*/*a*.

For hybrid parameters, such as slope, although it appears frequently in the theoretical analysis of the real contact in thermal contact resistance modeling[41], it is not employed in the present model.

The width of the model *H*_1_ is in the same order with the sampling length during surface profile measurement, and the height of the model *L*_1_ and *L*_2_ are typical dimensions of real structure.

The effective thermal conductivity of the micro-contact rectangle *k*′ is defined as[42]:

$$\frac{2}{k^{\prime }}=\frac{1}{k_{1}}+\frac{1}{k_{2}}$$ (3)

where *k*_1_ and *k*_2_ are temperature-dependent thermal conductivity of contact body 1 and 2 respectively.

### Solution of the model

#### Finite element algorithm

Finite element method is adopted here to solve the steady state heat transfer problem with micro-contact rectangles. The governing equations on the domain Ω bounded by ∂Ω can be expressed as:

∇⋅(k(T)∇T)=0onΩ (4)

where ∇ denotes the gradient operator, **k**(*T*) is a symmetric matrix of thermal conductivity coefficients which may vary with temperature *T*.

The boundary conditions of the problem are:

$$\begin{array}{l} T=\bar{T}\;\mathrm{on}\;\partial \Omega_{1}\\ n\cdot q=\bar{q}\;\mathrm{on}\;\partial \Omega_{2}\\ n\cdot q=h\left(T-T_{\infty }\right)\;\mathrm{on}\;\partial \Omega_{3}\\ n\cdot q=\varepsilon \sigma \left(T^{4}-T_{\infty }^{4}\right)\;\mathrm{on}\;\partial \Omega_{4} \end{array}$$ (5)

where $\sum_{i=1}^{4}\partial \Omega_{i}=\partial \Omega$, ∂Ω_*i*_∩∂Ω_*j*_ = ∅ (*i*, *j* = 1,2,3,4,*i* ≠ *j*). **q** is the heat flux, $\bar{T}$ and $\bar{q}$ are the prescribed temperature and heat flux respectively, *T*_∞_ is ambient temperature, **n** is the outward unit normal to the boundary ∂Ω. *h* denotes convective heat transfer coefficient, *ε* is the emissivity, and *σ* is the Stefan-Boltzmann constant (about 5.67×10^−8^ Wm^-2^K^-4^).

After space discretization, on a typical finite element Ω^*e*^, the weak form of the governing equation is given by:

$$\int_{\Omega^{e}}v^{e}\left(\nabla \cdot \left(k^{e}\nabla T^{e}\right)\right)d\Omega =0$$ (6)

where *v*^*e*^ is the scalar weighting function, and superscript *e* denotes element.

Integrating by parts the terms associated with the gradient in Eq (6) leads to:

$$\int_{\Omega^{e}}\left(\nabla v^{e}k^{e}\nabla T^{e}\right)d\Omega +\oint_{\partial \Omega^{e}}v^{e}\left(n^{e}\cdot q^{e}\right)d\Gamma =0$$ (7)

where **n**^*e*^ is the outward unit normal to the boundary ∂Ω^*e*^.

The temperature field of element *e* is assumed to be:

$$T^{e}=N_{T}^{e}T^{e}$$ (8)

where $N_{T}^{e}$ denotes the expansion basis or shape functions, **T**^*e*^ denotes listings of nodal temperatures for element *e*. In order to obtain a Galerkin style formulation, the weighting functions *v*^*e*^ is taken as:

$$v^{e}={\left(N_{T}^{e}\right)}^{T}$$ (9)

Substituting Eq (8) and Eq (9) into Eq (7), and considering boundary conditions in Eq (5), the following finite element equations can be obtained:

$$\left(K_{0}^{e}+K_{3}^{e}+K_{4}^{e}\right)T^{e}=F_{2}^{e}+F_{3}^{e}+F_{4}^{e}$$ (10)

where $K_{0}^{e}$, $K_{3}^{e}$, $K_{4}^{e}$, $F_{2}^{e}$, $F_{3}^{e}$ and $F_{4}^{e}$ are given by:

$$K_{0}^{e}=\int_{\Omega^{e}}{\left(\nabla N_{T}^{e}\right)}^{T}k^{e}\left(\nabla N_{T}^{e}\right)d\Omega$$ (11)

$$K_{3}^{e}=\int_{\partial \Omega_{3}^{e}}h{\left(N_{T}^{e}\right)}^{T}N_{T}^{e}d\Gamma$$ (12)

$$K_{4}^{e}=\int_{\partial \Omega_{4}^{e}}{\left(N_{T}^{e}\right)}^{T}\varepsilon \sigma {\left(T^{e}\right)}^{3}N_{T}^{e}d\Gamma$$ (13)

$$F_{2}^{e}=-\int_{\partial \Omega_{2}^{e}}{\left(N_{T}^{e}\right)}^{T}\bar{q}\;d\Gamma$$ (14)

$$F_{3}^{e}=\int_{\partial \Omega_{2}^{e}}{\left(N_{T}^{e}\right)}^{T}hT_{\infty }\;d\Gamma$$ (15)

$$F_{4}^{e}=\int_{\partial \Omega_{4}^{e}}{\left(N_{T}^{e}\right)}^{T}\varepsilon \sigma T_{\infty }^{4}\;d\Gamma$$ (16)

After assembling all the finite element equation, and imposing the prescribed temperature boundary conditions on ∂Ω_1_, the whole temperature field and heat flux field of the model can be obtained. Then the numerical results of thermal contact resistance can be given by:

$$R=\frac{{\bar{T}}_{\mathrm{top}}-{\bar{T}}_{\mathrm{bot}}}{\left(q_{\mathrm{top}}+q_{\mathrm{bot}}\right)/2}$$ (17)

where ${\bar{T}}_{\mathrm{top}}$ and ${\bar{T}}_{\mathrm{bot}}$ denote the average temperature at the high temperature side and low temperature side of the interface respectively, and *q*_top_ and *q*_bot_ denote the heat flux at the high temperature side and low temperature side of the interface respectively.

For high-temperature thermal contact resistance numerical simulation, the material properties such as thermal conductivity varies with temperature, and nonlinear radiation heat transfer may be taken into consideration, and these make the problem to be nonlinear, which needs iterative solution method to solve the finite element equations. The Picard iteration method is employed here and the convergence criterion is given by:

$$\frac{\left\|T^{\left(n\right)}-T^{\left(n-1\right)}\right\|}{\left\|T^{\left(n\right)}\right\|}\leq \delta_{T}$$ (18)

where *δ*_*T*_ is temperature convergence tolerance, and the superscript “*n*” denotes the iteration number.

#### Implementation of interface pressure and temperature

Although basic geometry parameters can be determined through the approach given in subsection 2.2, it is noted that these parameters just represent the initial contact status with zero contact pressure. After external load is applied to the two bodies, with the increment of the interface contact pressure, both the width of the micro-contact rectangle *a* and spacing of the rectangle *b* will be changed. It is obvious that real contact area increases with the increment of applied load, which implies that *a* increases and *b* decreases. Many models had been proposed to describe the relationship between real contact area and nominal contact area based on the deformation mechanism of the micro-contact [43], such as pure elastic deformation, elastoplastic deformation and pure plastic deformation. It is noted that most of these models are semi-empirical, which implies that some of the parameters of the model need to be determined by experiments. In this circumstance, with the consideration of high temperature at the interface, pure plastic deformation is adopted here. Song and Yovanovich proposed that for isotropic surfaces undergoing plastic deformation the relationship between real contact area and nominal contact area can be given by[44]:

$$\frac{A_{r}}{A_{n}}=\frac{P}{H_{c}}$$ (19)

where *A*_r_ and *A*_n_ denote the real contact area and nominal contact area respectively, and *P* is the nominal contact pressure at the interface, *H*_c_ is the appropriate micro-hardness value of the softer surface in contact, and can be given by:

$$\frac{P}{H_{c}}={\left(\frac{P}{c_{1}{\left(1.62R_{q}/m\right)}^{c_{2}}}\right)}^{\frac{1}{1+0.071c_{2}}}$$ (20)

where *m* is the absolute average asperity slope, *R*_q_ is the root-mean-squared roughness. *c*_1_ and *c*_2_ are experimentally determined parameters.

It can be seen from Eq (20) that *A*_r_/*A*_n_ is the function of nominal contact pressure *P* and surface roughness parameters *R*_q_ and *m*, all the other factors are included in the two coefficients *c*_1_ and *c*_2_. As interface temperature in the previous investigations is not that high, temperature effect is not that important, while in the present research, interface temperature is as high as 500°C, and temperature effect becomes dominant, then we propose a new empirical relationship here:

$$\frac{P}{H_{c}}=c_{0}+c_{1}\frac{P}{E_{0}^{\prime }}+c_{2}T_{\mathrm{int}}+{\left(\frac{P}{E_{0}^{\prime }}\right)}^{c_{3}}T_{\mathrm{int}}^{c_{4}}$$ (21)

where *c*_0_, *c*_1_, *c*_2_, *c*_3_ and *c*_4_ are experimentally determined dimensionless parameters. As surface roughness parameters have already considered in the geometry modelling of the finite element model, they didn’t appear in Eq (21), instead, average interface temperature *T*_int_ appears. The effective elastic modulus $E_{0}^{\prime }$ is defined as[41]:

$$\frac{1}{E_{0}^{\prime }}=\frac{1-\nu_{1}^{2}}{E_{1}}+\frac{1-\nu_{2}^{2}}{E_{2}}$$ (22)

where *E*_1_ and *E*_2_ are elastic modulus of contact body 1 and 2 at room temperature respectively, and *ν*_1_ and *ν*_2_ are Poisson’s ratio respectively.

It is noted that the plane heat transfer model is adopted here, then the relationship between *A*_r_/*A*_n_ and spacing ratio *b*/*a* can be given by:

$$\frac{A_{r}}{A_{n}}=\frac{a}{a+b}=\frac{1}{1+b/a}$$ (23)

After determine the dimensionless parameters *c*_0_, *c*_1_, *c*_2_, *c*_3_ and *c*_4_ in Eq (21), through Eq (19) and Eq (23) the spacing ratio *b*/*a* can be given by:

$$\frac{b}{a}=\frac{1}{c_{0}+c_{1}\frac{P}{E_{0}^{\prime }}+c_{2}T_{\mathrm{int}}+{\left(\frac{P}{E_{0}^{\prime }}\right)}^{c_{3}}T_{\mathrm{int}}^{c_{4}}}-1$$ (24)

Eq (24) gives the relationship between the spacing ratio and the nominal contact pressure and average interface temperature, and spacing ratio is the key parameter to the numerical results of high-temperature thermal contact resistance. Then the flowchart of the numerical simulation of high-temperature thermal contact resistance is given below:

1. Input geometric parameters *R*_q1_, *R*_q2_, *S*_m1_, *S*_m2_, *L*_1_, *L*_2_, *H*_1_ and nominal pressure *P*, and the prescribed temperature boundary conditions.
2. Build geometry model with parameters given by Eq (1), Eq (2) and Eq (24).
3. Mesh the model, apply boundary conditions and solve the total assembled finite element equations to obtain the temperature field of the model, and then compute the thermal contact resistance denoted by *R*^(0)^ through Eq (17) and average interface temperature $T_{\mathrm{int}}^{\left(0\right)}$.
4. Update the finite element model with updated spacing ratio given by Eq (24) with the present interface temperature, and solve the total assembled finite element equations to obtain the updated temperature field, then compute the updated thermal contact resistance by *R*^(*n*)^ and average interface temperature $T_{\mathrm{int}}^{\left(n\right)}$.
5. If $\left|\frac{R^{\left(n\right)}-R^{\left(n-1\right)}}{R^{\left(n\right)}}\right|\leq \delta_{R}$, then the present *R*^(*n*)^ is the computed thermal contact resistance, otherwise, return to Step 4 to try another iteration. *δ*_*R*_ is temperature convergence tolerance, and the superscript “*n*” denotes the iteration number.

Through this iteration solution procedure, the effect of surface roughness is implemented through the length of the micro-contact rectangle *a*, and the effects of both applied pressure *P* and average interface temperature *T*_int_ are also implemented through Eq (24). This iteration procedure also implies a type of thermo-mechanical coupling problem with thermal contact resistance[45]. Other than the conventional thermomechanical coupling problems, thermal contact resistance plays an important role here.

#### Implementation of thermal interface material

The previous sections give the numerical simulation of high-temperature thermal contact resistance, when thermal interface materials (TIMs) are used to reduce thermal contact resistance, special attention should be paid. Generally, TIM is always chosen to be soft material with high thermal conductivity. For high temperature applications, TIM also should be heat resistant. The physical mechanism of the TIM on reducing TCR is that, under certain applied pressure the soft TIM can fill the micro-contact cavities at the interface to increase heat transfer through heat conduction. The higher the applied pressure is, the more the cavities will be filled. When all the cavities are full, no more TIM can be pushed into the cavities with the increment of the applied pressure. If the TIM is too thick, it will be blocked at the interface, and then the total TCR might be increased.

In the present numerical simulation, when TIM is adopted to reduce the thermal contact resistance, it is assumed that the TIM will fill all the cavities at the interface, as TIM is always very soft. Based on this assumption, the heat transfer mechanism of the interface cavities is heat conduction with TIM, rather than radiation through cavities. Meanwhile, TCR with TIM at the interface may vary with the thermal conductivity of the TIM, the equivalent surface roughness of the interface and the thickness of the TIM, and this will be parametrically investigated in the next section. It should be pointed out that, in real applications, although the TIM might be very soft, there still could have extra thermal contact resistance between the TIM and the two specimens. As it is very hard and unnecessary to give a quantitative description of this effect, it is ignored here.

## Numerical examples and discussions

In this section, the complete numerical simulation approach is illustrated. The determination of parameters *c*_0_, *c*_1_, *c*_2_, *c*_3_ and *c*_4_ is crucial to the whole finite element modeling of high-temperature thermal contact resistance, it relates the real contact area and the thermal contact resistance. Through numerical results given by the previously proposed approach, we can determine the parameters in Eq (24) with the experimental results under the following routines.

1. Conduct thermal contact resistance tests, to determine TCR *R*^EXP^ under different interface temperature $T_{\mathrm{int}}^{\mathrm{EXP}}$ and applied pressure *P* with practical surface roughness specimens.
2. Conduct numerical simulation of thermal contact resistance with numerical approach described in Section 2, to determine TCR *R*^FEA^ under different interface temperature $T_{\mathrm{int}}^{\mathrm{FEA}}$ and spacing ratio *b*/*a* with the same surface roughness during experimental investigations.
3. Compare the experimental and numerical results of thermal contact resistance, under the same interface temperature and thermal contact resistance, a discrete mapping between the spacing ratio *b*/*a* and average interface temperature *T*_int_ and applied pressure *P* can be established.
4. With the relationship between *b*/*a* and *T*_int_ and *P* obtained previously, the regression values of *c*_0_, *c*_1_, *c*_2_, *c*_3_ and *c*_4_ in Eq (24) can be given through nonlinear data fitting.
5. After obtaining the relationship between *b*/*a* and *T*_int_ and *P*, the finite element simulation approach can be used to determine high-temperature thermal contact resistance under any circumstances.

### Experimental results

In order to determine the key parameters of the finite element model in Eq (24), experimental tests should be conducted firstly[46]. The test facility is based on INSTRON 8874 high-temperature material testing machine and consists of a test column, a loading system, a heating and cooling unit and a temperature measurement system. The axial temperature distribution in the specimens is recorded by several K-Type thermocouples, and the axial heat flux as well as the temperature jump across the interface can be obtained based on the recorded temperature distribution with Fourier’s law. Cylindrical specimens (30 mm diameter and 40 mm length) were made from GH600 and three-dimensional braid C/C composite. The conductivity of C/C composite material is assumed to be a constant value of 66.1 Wm^-1^K^-1^ [46], and the conductivity of superalloy GH600 varies with temperature[45]. The surface roughness of the specimen is tested by Talysurf 5P-120 surface topography instrument made by Rank Taylor Hobson Company in the State Key Laboratory of Tribology (SKLT) at Tsinghua University, and the root-mean-squared roughness of the C/C and the GH600 specimens are 32.7 *μ*m and 55.4 *μ*m respectively. The mean spacing between profile peaks at the mean line of contact surface of the C/C and the GH600 specimens are 258 *μ*m and 656 *μ*m respectively. The experimental results of the high-temperature thermal contact resistance with different average interface temperatures and contact pressures are given (Table 1). It is noted that 0 applied contact pressure in Table 1 implies that only dead weight of the specimen is applied at the interface.

**Table 1:** Experimental results of the high-temperature thermal contact resistance.

Average interface temperature (K)	Applied contact pressure (MPa)	Thermal contact resistance (Km^2^/W)
499	0	1.08E-03
543	0	1.02E-03
583	0	9.75E-04
622	0	9.67E-04
673	0	9.36E-04
718	0	8.90E-04
758	0	8.62E-04
796	0	7.93E-04
824	0	7.67E-04
493	8.5	3.93E-04
530	8.5	3.74E-04
583	8.5	3.50E-04
636	8.5	3.30E-04
678	8.5	3.22E-04
723	8.5	3.17E-04
751	8.5	3.14E-04
784	8.5	3.12E-04
487	17	3.59E-04
540	17	3.33E-04
605	17	3.04E-04
664	17	2.84E-04
711	17	2.62E-04
753	17	2.49E-04
787	17	2.45E-04
817	17	2.31E-04

### Numerical results

**Fig 4:**
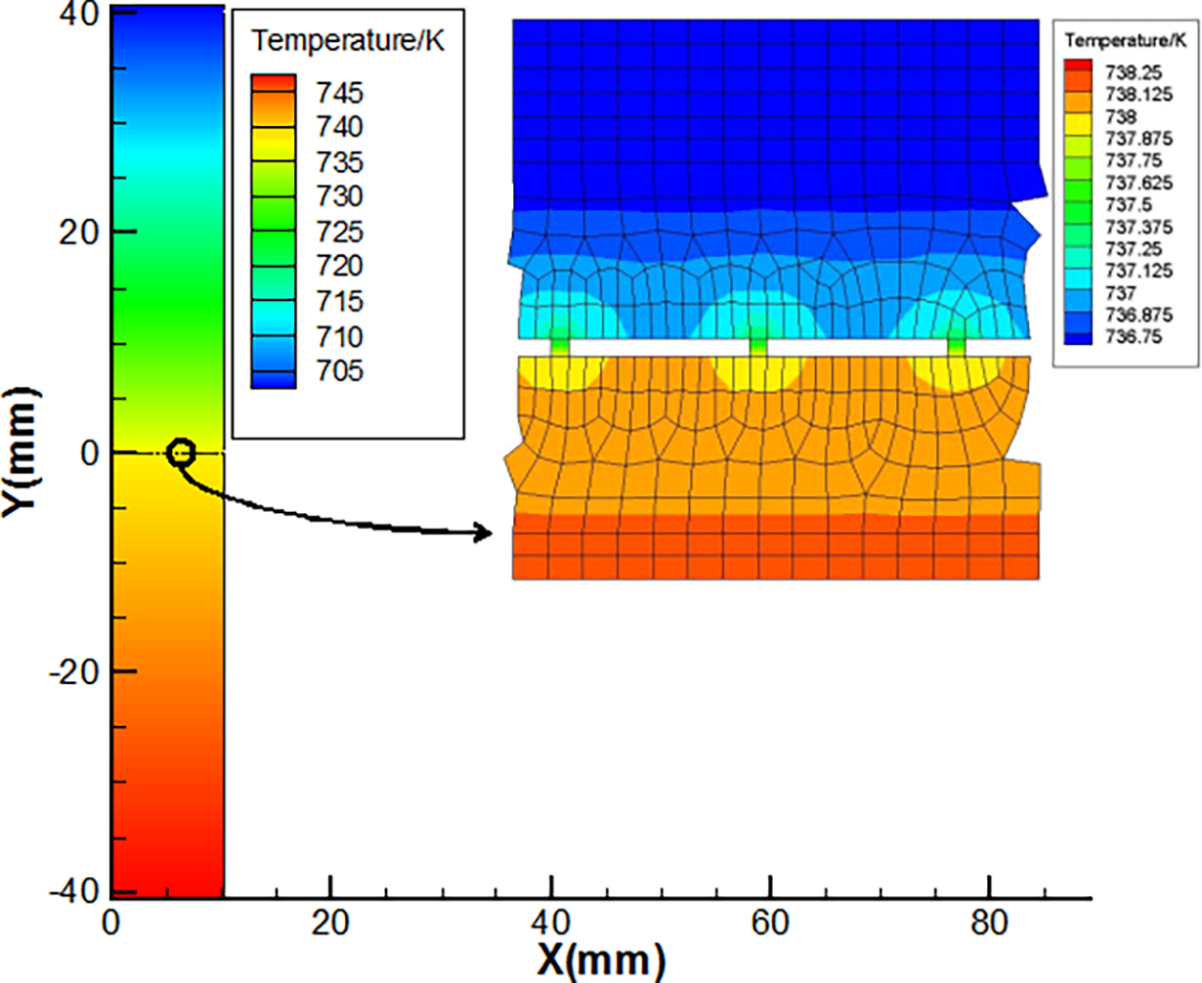
Temperature field of the model with radiation heat transfer at the interface.

Firstly, a typical temperature field of the finite element model is illustrated. The material used the same as in the experiments described in subsection 3.1, and the geometry parameters are also given below (Table 2). The top and bottom boundaries are applied with prescribed temperature of 700 K and 750 K respectively, other outer boundaries are assumed to be adiabatic, radiation boundary conditions are applied on the surfaces of the cavities to take into account the effect of radiation heat transfer between the two bodies across the cavities, and the surface emissivity of C/C material and superalloy GH600 material are assumed to be 0.9 and 0.7 respectively. The air between the microcontact spots are considered as conduction medium with a temperature-dependent thermal conductivity. The temperature field of the model is given (Fig 4). A total of 38912 nodes and 38242 elements are used in the finite element model. Convergence test also show the reliability of the present numerical results.

**Table 2:** Input parameters of the numerical model.

Length of the model, *H*_1_ (mm)	10.0
Height of specimen 1, *L*_1_ (mm)	40.0
Height of specimen 2, *L*_2_ (mm)	40.0
Width of the microcontact rectangular, *a* (mm)	53.1E-3
Height of the microcontact rectangular, *t* (mm)	53.1E-3
Spacing of the microcontact rectangular, *b* (mm)	545E-3

It can be seen from Fig 4 that due to the existence of the gaps, although radiation heat transfer may occurs through the gaps, temperature jump phenomena appears at the real contact interface, and it is determined through the difference between average temperature of the upper surface and lower surface. After computing the average heat flux of the two contact bodies, then the numerical thermal contact resistance can be obtained with Eq (17). The results are 4.75×10^−5^ Km^2^/W with interface radiation and 4.82×10^−5^ Km^2^/W without interface radiation, and it is clear that interface radiation has little contribution to the heat transfer through the contact surface under this circumstance. Numerical results also show that if the spacing ratio is very large, which implies that the applied pressure is very small, and then heat transfer through the gaps at high temperature conditions will be non-negligible.

**Fig 5:**
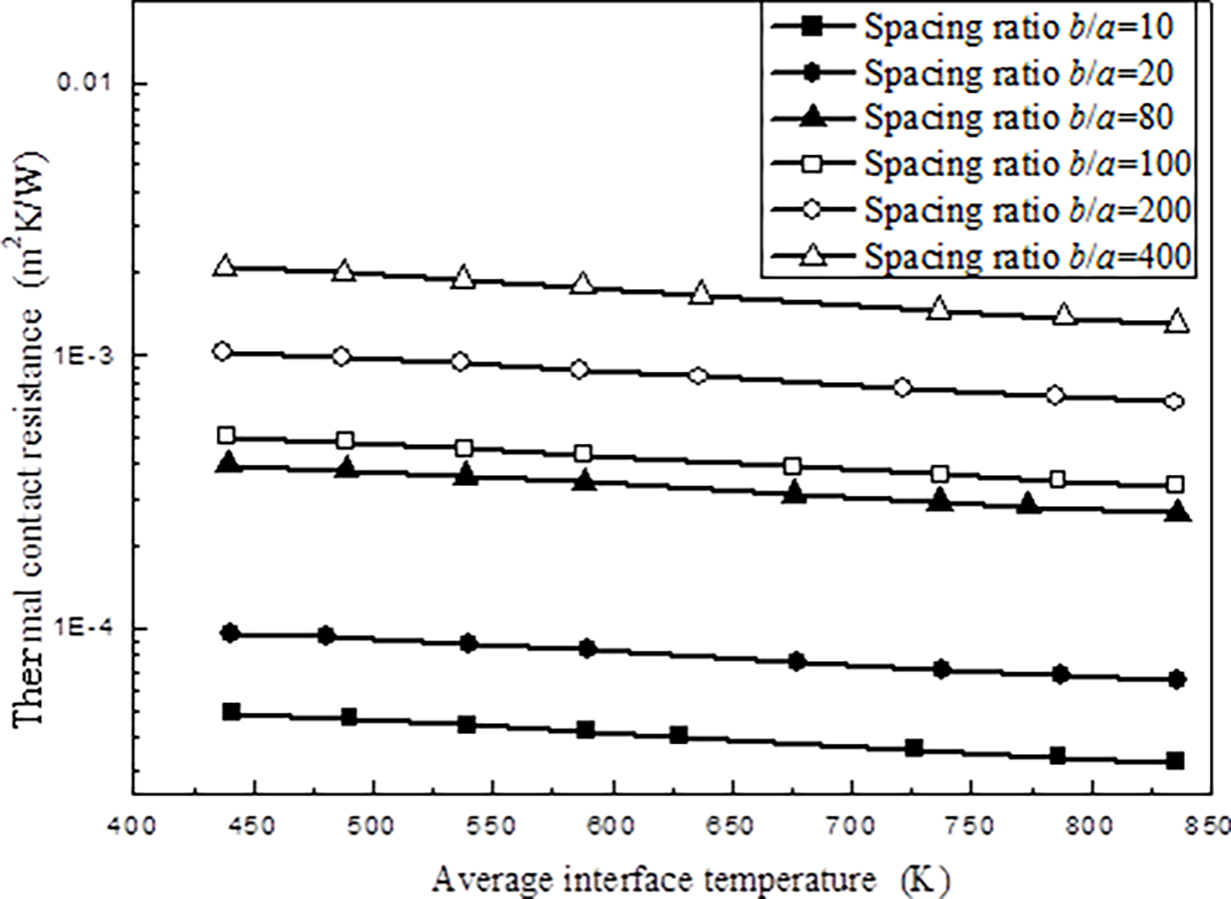
Variations of thermal contact resistance with different average interface temperature *T*_int_ and spacing ratio *b*/*a*.

During the following numerical simulations, the micro-contact width *a* is kept the same as *a* = 10 *μ*m, and the variations of the thermal contact resistance with average interface temperature *T*_int_ and spacing ratio *b*/*a* with the present numerical approach are given below (Fig 5).

Fig 5 clearly shows that, with the increment of the average interface temperature thermal contact resistance decrease slowly, due to the enhancement of the interface radiation heat transfer. At the same time, larger spacing ratio implies that less micro-contact occurs at the interface under the same length, which corresponds larger thermal contact resistance. Numerical results given in Fig 5 will be used to establish the relationship between applied pressure, average interface temperature and spacing ratio.

### Comparisons and regression

**Fig 6:**
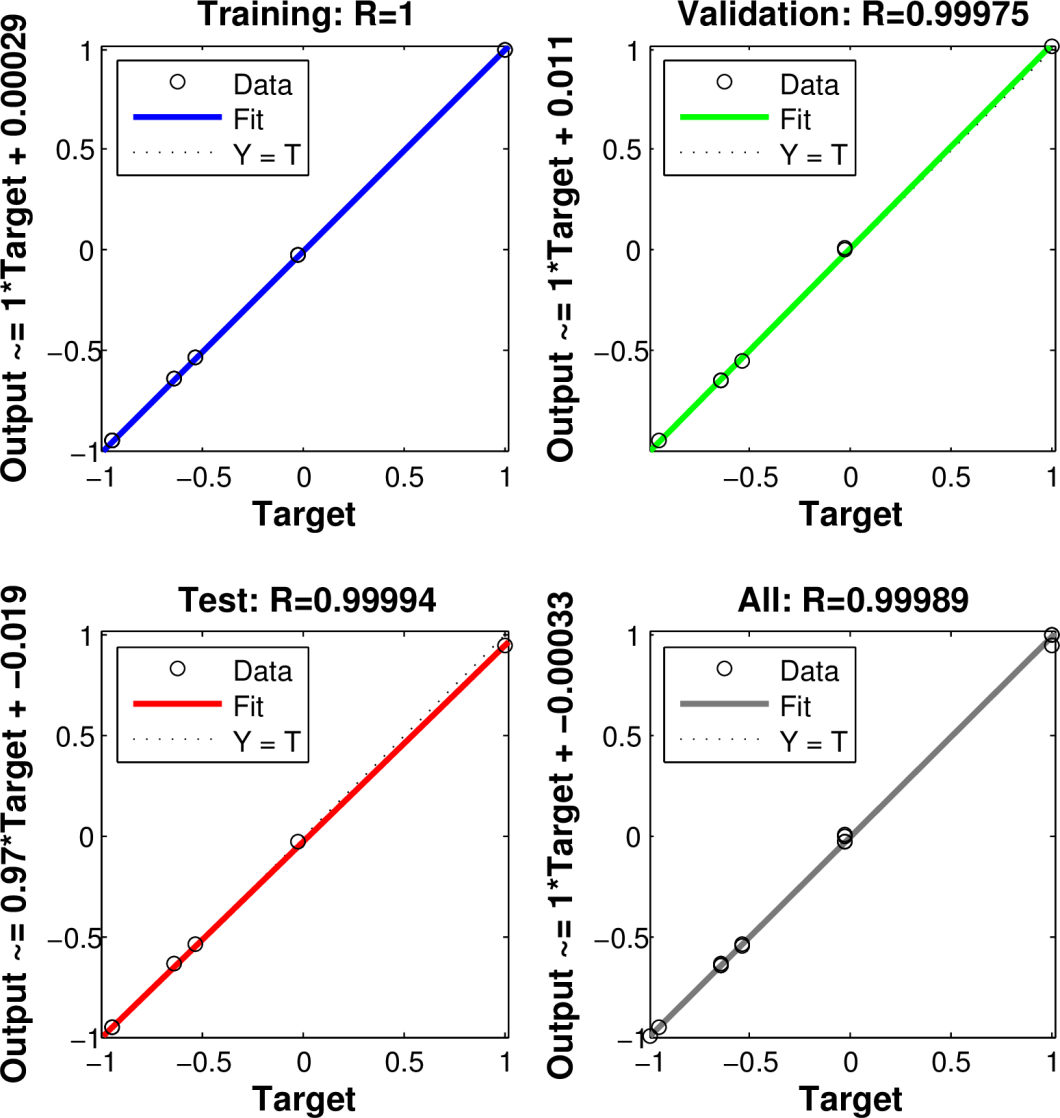
Regressions of the training and test samples.

**Fig 7:**
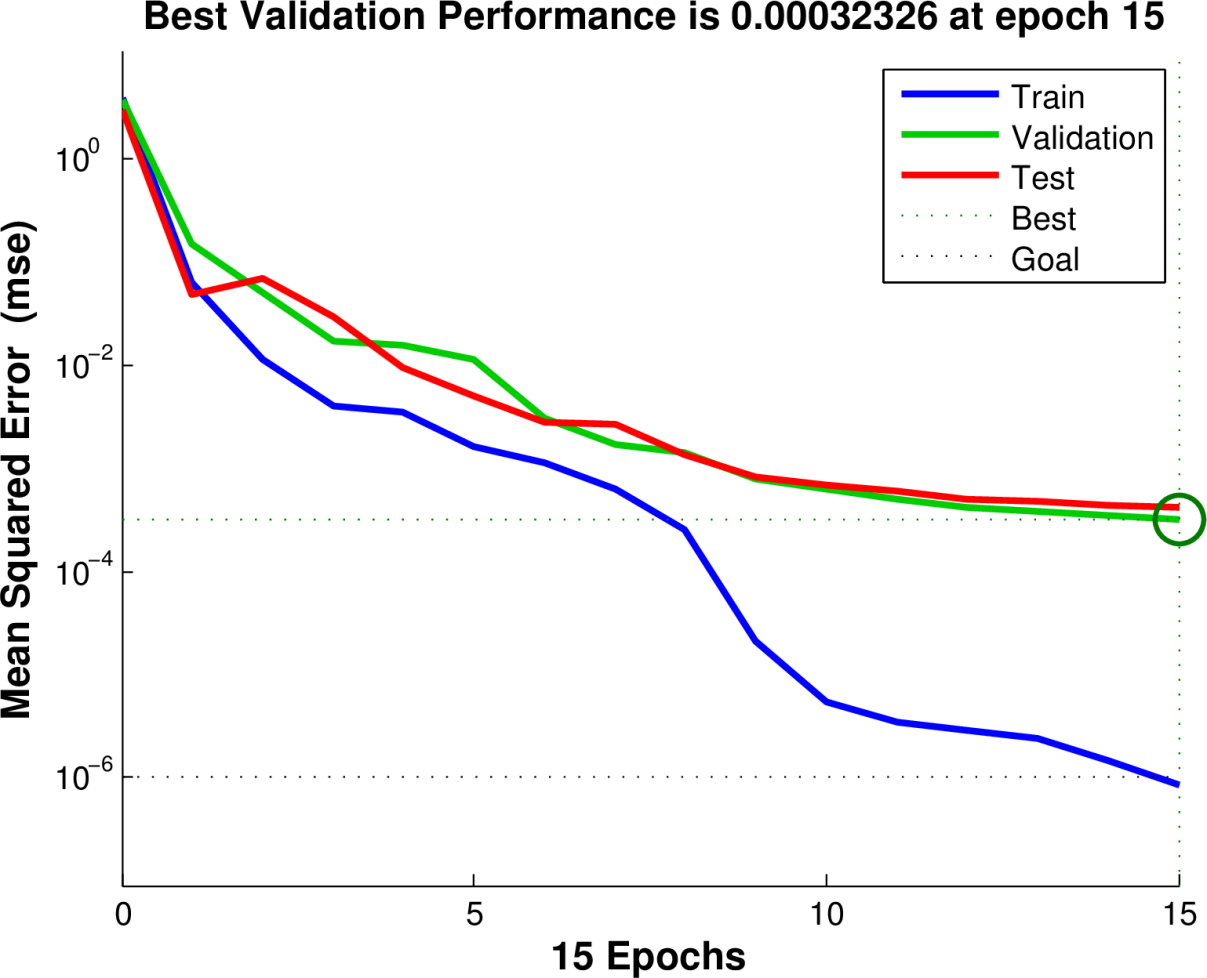
Training process of the neural network.

After obtaining experimental results ($T_{\mathrm{int}}^{\mathrm{EXP}}$, *R*^EXP^, *P*) and numerical results ($T_{\mathrm{int}}^{\mathrm{FEA}}$, *R*^FEA^, *b*/*a*), the relationship between average interface temperature *T*_int_, applied pressure *P* and spacing ratio *b*/*a* can be determined through Neural Network Method. Neural networks are composed of simple elements operating in parallel, and these elements are inspired by biological nervous systems. Typically, neural networks need to be trained, so that a particular input leads to a specific target output. In the present paper, the numerical results are considered as the training samples, and ($T_{\mathrm{int}}^{\mathrm{FEA}}$, *R*^FEA^) are taken as training inputs, and the spacing ratio *b*/*a* is the training output. At the same time, for experimental results, ($T_{\mathrm{int}}^{\mathrm{EXP}}$, *R*^EXP^) are taken as test inputs, and test outputs are corresponding spacing ratio *b*/*a* at the same ($T_{\mathrm{int}}^{\mathrm{FEA}}$, *R*^FEA^) combination. Through this process, for a given experimental data point ($T_{\mathrm{int}}^{\mathrm{EXP}}$, *R*^EXP^, *P*), using ($T_{\mathrm{int}}^{\mathrm{FEA}}$, *R*^FEA^) as input, we can obtain corresponding *b*/*a* as output, and data point ($T_{\mathrm{int}}^{\mathrm{EXP}}$, *R*^EXP^, *P*, *b*/*a*) can be confirmed. Then, the relationship between interface temperature *T*_int_, applied pressure *P* and spacing ratio *b*/*a* are established with thermal contact resistance *R* as internal variable. The regressions of the training and test samples are also given (Fig 6), and Fig 7 shows the training process of the neural network.

**Fig 8:**
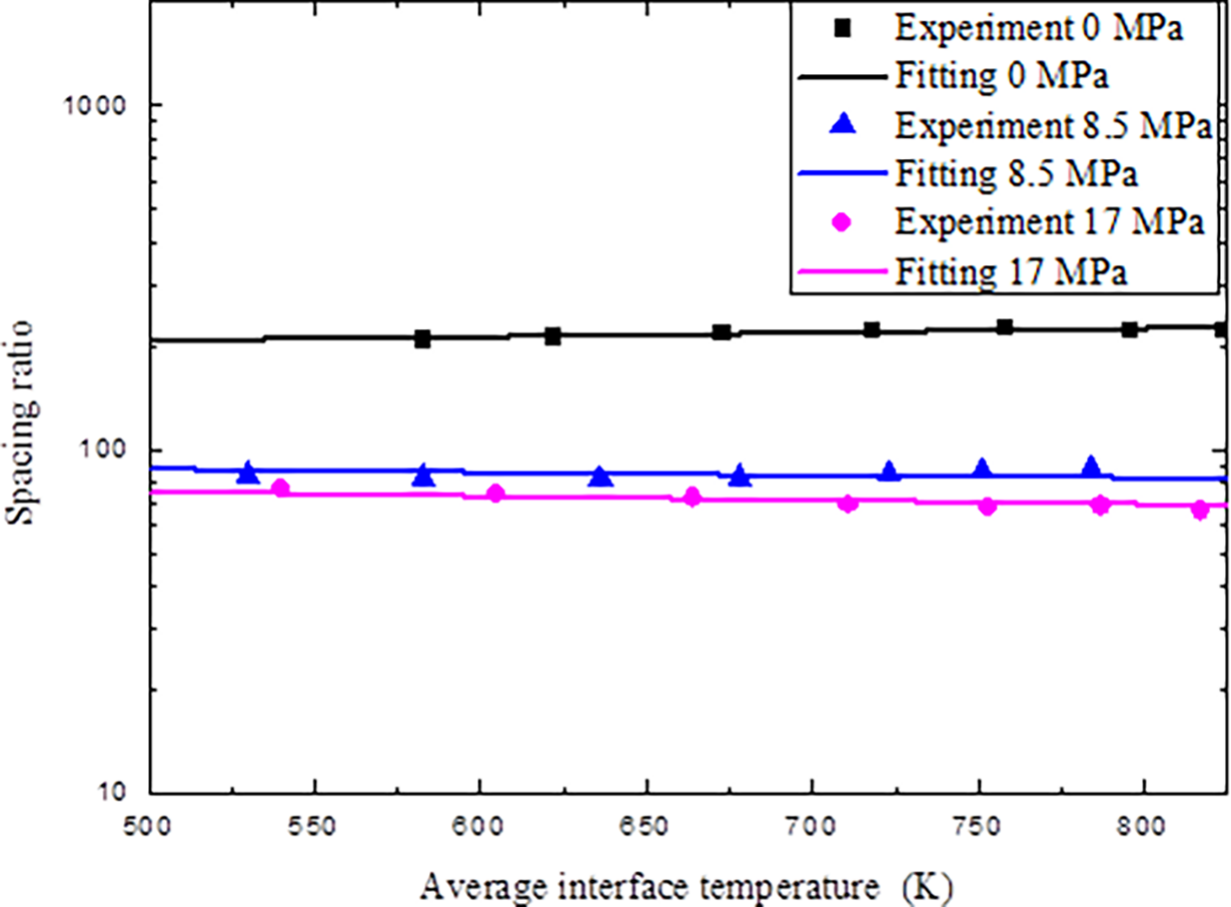
Comparisons of experimental data and fitting data.

It can be seen from Figs 6 and 7 that the present neural network worked very well. Once the relationship between average interface temperature *T*_int_, applied pressure *P* and spacing ratio *b*/*a* are determined, we can determine the coefficients in Eq (24) using nonlinear regression with the Levenberg-Marquardt algorithm for nonlinear least squares to compute non-robust fits. Results show that *c*_0_ = 0.00541, *c*_1_ = 0.563, *c*_2_ = 0.229, *c*_3_ = -13.876, and *c*_4_ = −3.444E-4. The comparisons of the original data and fitting data about the spacing ratio are given (Fig 8). It can be seen from Fig 8 that the original data compare very well with the fitting data on spacing ratio.

**Fig 9:**
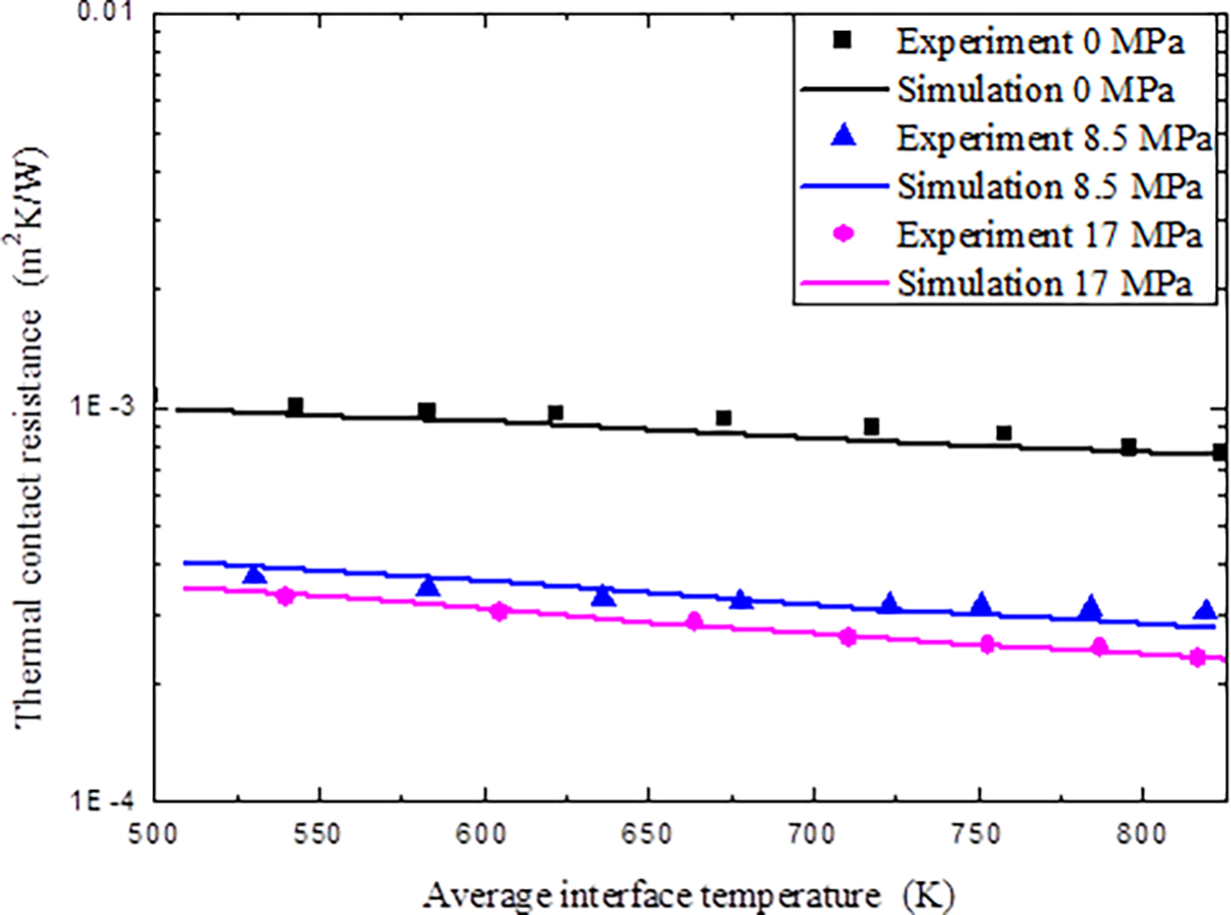
Comparisons of experiment and numerical simulation on thermal contact resistance.

Furthermore, the comparisons of the experimental results given in section 3.1 and numerical results based on the fitting spacing ratio of the thermal contact resistance are given (Fig 9). It can be seen that the numerical results compared well with the experimental results, which show that the present numerical approach can be used to simulate high-temperature thermal contact resistance.

## Discussions

Generally, thermal contact resistance is considered as the function of surface roughness, applied pressure and interface temperature. In the present numerical model, the effect of surface roughness and applied pressure are taken into account through micro-contact width *a* and spacing *b*. The micro-contact width *a* is assumed to be a constant 10 *μ*m in the following parametric study, the higher the applied pressure is, the smaller the spacing *b* will be, and the spacing ratio will be smaller in the meantime which implies that more microspots participate into contact. Based on the previously obtained relationship between *P*, *T*_int_ and spacing ratio *b*/*a*, the effects of some key parameters of the finite element model are numerically investigated with the present multi-point contact model.

**Fig 10:**
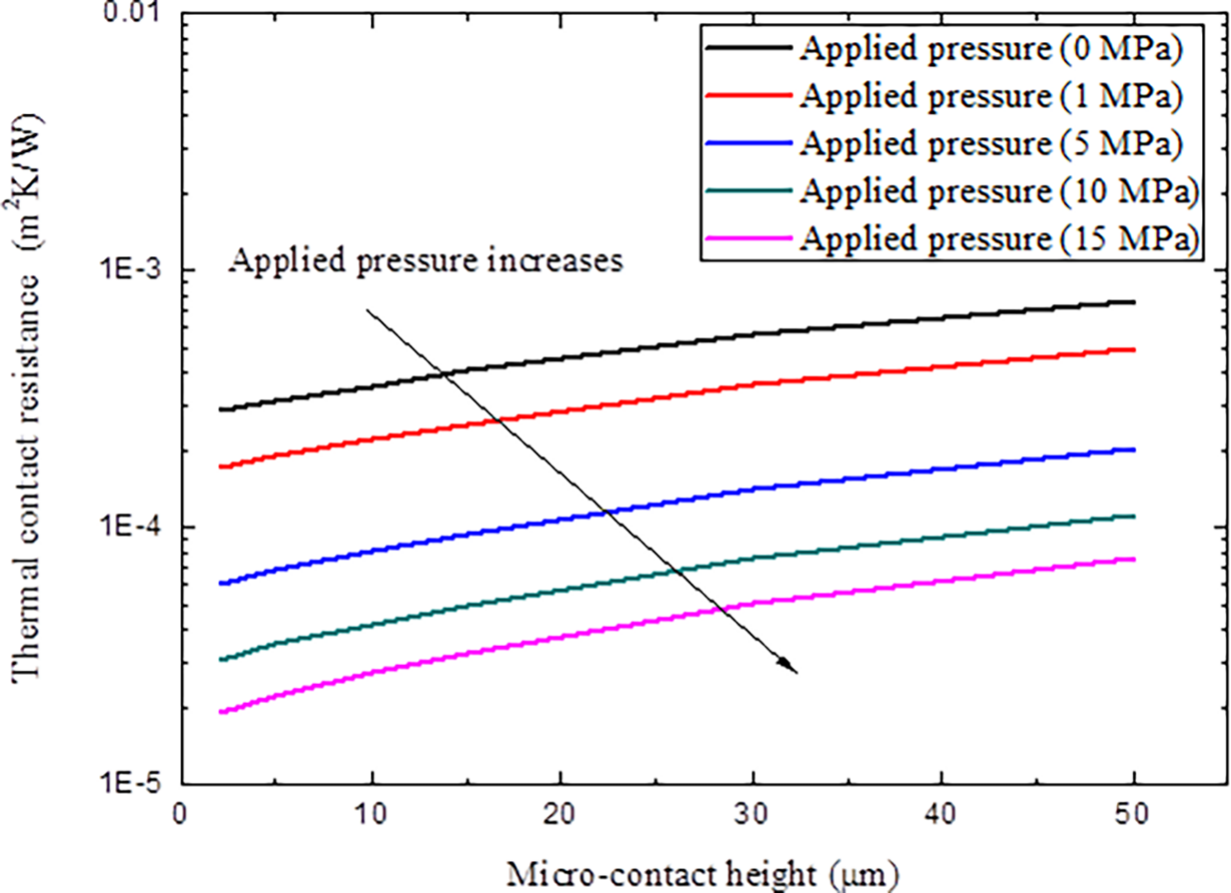
Variations of thermal contact resistance with different micro-contact height *t* and applied pressure *P*.

Keeping the upper boundary and lower boundary of the specimens as 700 K and 750 K unchanged, then the variations of thermal contact resistance with different micro-contact height *t* and applied pressure *P* are given in Fig 10.

**Fig 11:**
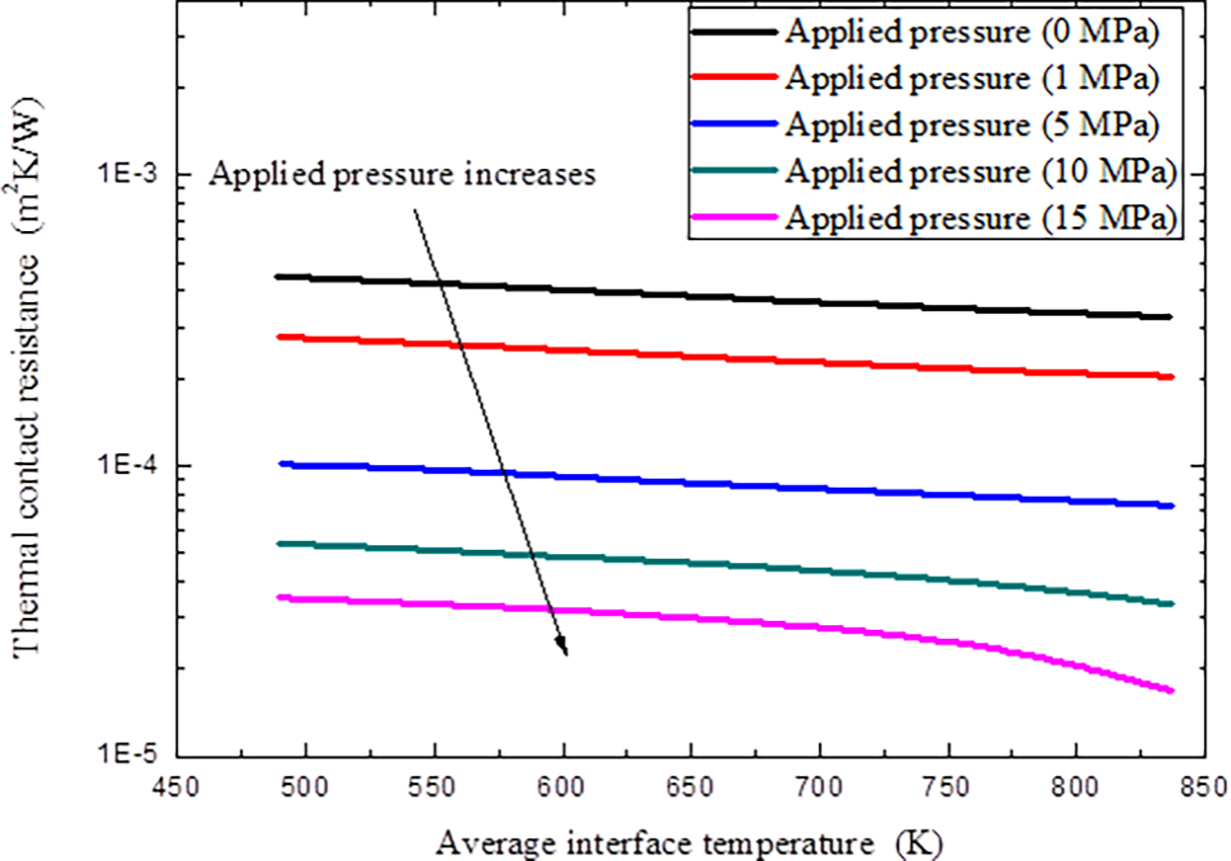
Variations of thermal contact resistance with different average interface temperature *T*_int_ and applied pressure *p*.

The variations of thermal contact resistance with different average interface temperature *T*_int_ and applied pressure *P* are given in Fig 11 under a constant micro-contact width *a* = 10 *μ*m and height *t* = 10 *μ*m.

**Fig 12:**
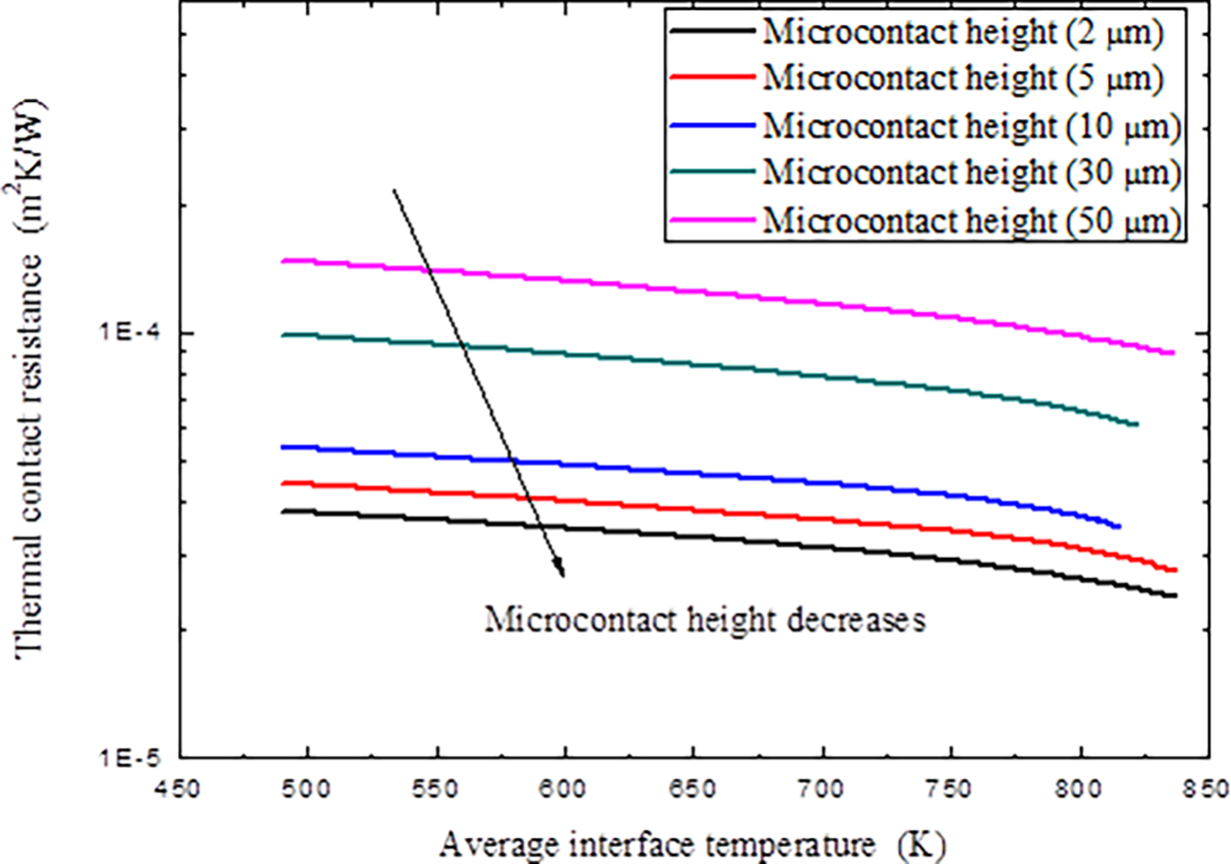
Variations of thermal contact resistance with different average interface temperature *T*_int_ and micro-contact height *t*.

The variations of thermal contact resistance with different average interface temperature *T*_int_ and micro-contact height *t* are given in Fig 12 under a constant applied pressure *P* = 10 MPa and a constant micro-contact width *a* = 10 *μ*m.

As can be seen from the results in Figs 10–12, thermal contact resistance decreases with the increment of applied pressure. As the contact pressure at the interface is increased, the contact asperities deform further in addition to new asperities coming into contact, which increases the amount of actual contact area and the spacing ratio decreases. The solid spot contribution to thermal contact conductance correspondingly increases. With the increment of microcontact height, thermal contact resistance increase, as large micro-contact height means coarse surface, also implies that little micro-spots can come into contact and results in difficulty in interface heat transfer. At the same time, with the increment of the interface temperature, thermal contact resistance decreased because of the increment of interface radiation heat transfer and material degradation under high temperatures, as high temperature always makes material softer to make micro-contact easier to occur. It is also noted that the thermal contact resistance decrement through temperature effect is not very obvious compared with the effects of applied pressure and microcontact height.

**Fig 13:**
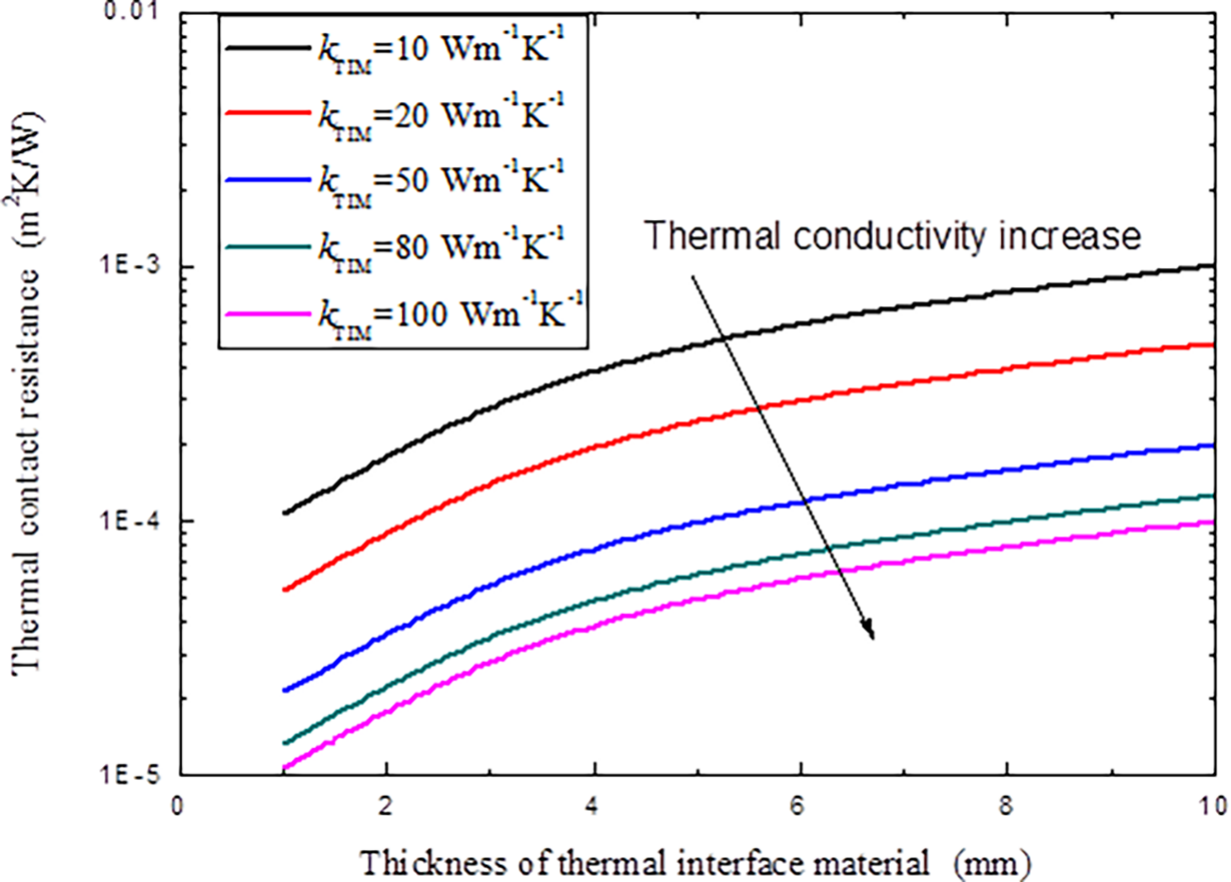
Variations of thermal contact resistance with different TIM thickness and thermal conductivity *k*_TIM_.

**Fig 14:**
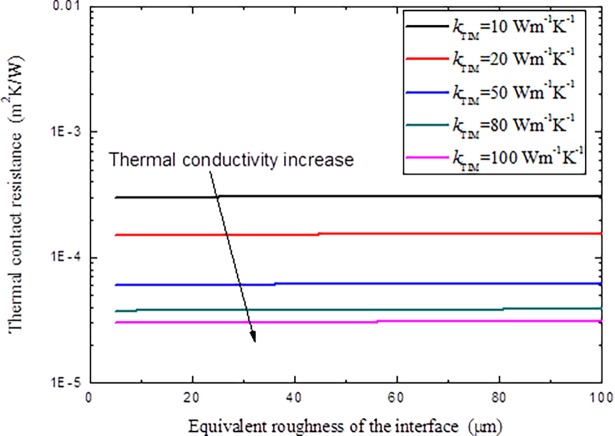
Variations of thermal contact resistance with different TIM thickness and equivalent roughness of the interface.

If TIM is adopted at the interface to reduce thermal contact resistance, the variations of the TCR with different TIM thermal conductivity, thickness and the equivalent surface roughness of the interface are given in Figs 13 and 14.

It is clear in Figs 13 and 14 that, higher thermal conductivity of the TIM implies that heat can pass the interface easier, which shows smaller TCR. For a given TIM, with the increment of the thickness of the TIM, TCR increases, as only a very small portion of the TIM can be pushed into the interface cavities, most of them will act as new obstacle to the heat transfer through the interface, the thicker the TIM is, the more serious this will be. But if the TIM is too thin, the TCR reduction effect is also depressing, so it is very necessary to determine a suitable TIM thickness for engineering applications. It is also noted that for a given TIM, the equivalent surface roughness of the interface has little influence on the TCR, as most of the contributions of the TCR origins from the redundant thickness of the TIM since it is assumed that the TIM will fill all the cavities during operation.

## Conclusions

The computational model and a finite element algorithm for high temperature thermal contact resistance simulation are established based on the multi-point contact theory, and the basic parameters of the computational model can be determined by surface roughness measurement and TCR experiments. The effect of the contact pressure is implemented to the finite element model through geometric parameter spacing ratio, which expressed by the function of contact pressure and average interface temperature, and the constant parameters within it is determined through Neural Network Method. Due to the thermomechanical coupling nature of the heat transfer problem, an iteration algorithm is also adopted to solve the finite element equations. The effect of the thermal interface material is also involved in the present numerical model through special treatment. The effects of the crucial parameters, such as contact pressure, average interface temperature and equivalent surface roughness, on the thermal contact resistance with and without TIM are also investigated with the proposed finite element model.

Numerical results show that, the present numerical results compare well with the experimental results on both spacing ratio and thermal contact resistance, which imply that the present finite element model and computational method can simulate high-temperature thermal contact resistance under different conditions, and could be an alternative approach to modeling high-temperature thermal contact resistance with and without thermal interface materials.
